# Ultrasound-Triggerable Coatings for Foley Catheter Balloons for Local Release of Anti-Inflammatory Drugs during Bladder Neck Dilation

**DOI:** 10.3390/pharmaceutics14102186

**Published:** 2022-10-13

**Authors:** Olga A. Sindeeva, Arkady S. Abdurashitov, Pavel I. Proshin, Alexey V. Kadrev, Oleg A. Kulikov, Boris M. Shaparov, Nikolay I. Sorokin, Valentin P. Ageev, Nikolay A. Pyataev, Aleksandr Kritskiy, Alexander Tishin, Armais A. Kamalov, Gleb B. Sukhorukov

**Affiliations:** 1 A.V. Zelmann Center for Neurobiology and Brain Rehabilitation, Skolkovo Institute of Science and Technology, Bolshoy Boulevard 30, 121205 Moscow, Russia; 2 Ultrasound Diagnostics Department, Medical Research and Educational Center, Lomonosov Moscow State University, 27 Lomonosovsky Ave., 119192 Moscow, Russia; 3 Diagnostic Ultrasound Division, Russian Medical Academy of Continuous Professional Education, 1 Barrikadnaya Str., 125445 Moscow, Russia; 4 Institute of Medicine, National Research Ogarev Mordovia State University, 68 Bolshevistskaya Str., 430005 Saransk, Russia; 5 Department of Urology and Andrology, Faculty of Fundamental Medicine, Medical Scientific and Educational Center, Lomonosov Moscow State University, 27 Lomonosovsky Ave., 119192 Moscow, Russia; 6 LLC Magnetic Drug Delivery, AMT & C Group, 4 Promyshlennaya Str., Troitsk, 108840 Moscow, Russia; 7 Siberian State Medical University, 2 Moskovskiy Trakt, 634050 Tomsk, Russia; 8 School of Engineering and Materials Science, Queen Mary University of London, Mile End Road, London E1 4NS, UK

**Keywords:** ultrasound-induced release, ultrasound-sensitive coatings, PLA coating, local drug delivery, microchamber arrays, anti-inflammatory drug, methylprednisolone sodium succinate, Prednol-L, Foley catheter, balloon dilation, bladder neck contracture

## Abstract

Bladder neck contracture (BNC) is a complication of the surgical treatment of benign and malignant prostate conditions and is associated with the partial or complete blockage of urination. Correction of this condition usually requires repeated surgical intervention, which does not guarantee recovery. Balloon dilation is a minimally invasive alternative to the surgical dissection of tissues; however, it significantly reduces the patient’s quality of life. Additional local anti-inflammatory treatment may reduce the number of procedures requested and increase the attractiveness of this therapeutic strategy. Here, we report about an ultrathin biocompatible coating based on polylactic acid for Foley catheter balloons that can provide localized release of Prednol-L in the range of 56–99 µg in the BNC zone under conventional diagnostic ultrasound exposure. Note that the exposure of a transrectal probe with a conventional gray-scale ultrasound regimen with and without shear wave elastography (SWE) was comparably effective for Prednol-L release from the coating surface of a Foley catheter balloon. This strategy does not require additional manipulations by clinicians. The trigger for the drug release is the ultrasound exposure, which is applied for visualization of the balloon’s location during the dilation process. In vivo experiments demonstrated the absence of negative effects of the usage of a coated Foley catheter for balloon dilation of the bladder neck and urethra.

## 1. Introduction

Bladder neck contracture (BNC) is one of the well-described complications of surgical treatment of benign and malignant prostate conditions [1]. This disease is characterized by the development of cicatricial deformation in the bladder neck area, leading to difficulty or complete blockage of urination. The etiologies and probability of BNC development are highly dependent on the primary surgical treatment [1,2].

**Figure 1:**
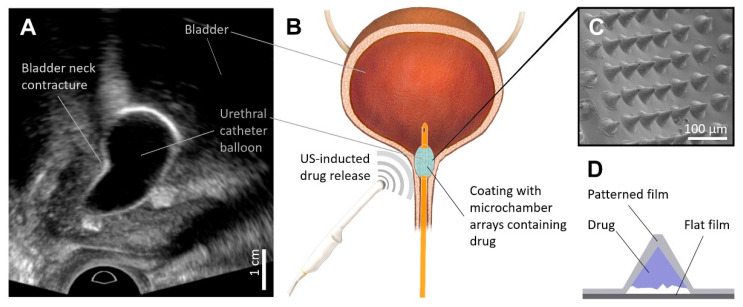
Gray-scale ultrasound imaging of bladder neck contracture balloon dilation (**A**). The proof of concept for the ultrasound-induced local delivery of the anti-inflammatory drug into the scar tissue during balloon dilation of bladder neck contracture (**B**). Typical SEM image of PLA-based coating with microchambers arrays (**C**). General scheme of microchamber structure (**D**).

The options for treatment techniques can vary from simple, office-based dilation procedures (Figure 1A) to more-invasive, complex abdominoperineal reconstructive surgery [1,3]. Transurethral resection is currently the main treatment option for BNC, but the recurrence rate after this procedure can reach up to 38% [4]. At the same time, balloon dilation [4,5] and self-dilation [6] can be promising and minimally invasive alternatives for the treatment of recurrent BNC [4,7,8,9]. However, this option is viable only for well-motivated patients because the treatment involves several procedures with an individually selected frequency and requires a great deal of tolerance and compliance [1]. Ultimately, many patients interrupted the course of treatment, likely due to its negative impact on their quality of life [6]. Reducing the number of procedures could make this therapeutic strategy more attractive to patients. Local injection of steroid [10], anti-inflammatory, anti-fibroblast, and anti-collagen [11] drugs in combination with other therapies has already shown its effectiveness. In this regard, the combination of balloon dilation with the local application of such drugs seems to be a rather promising strategy to reduce the restenosis number.

Local release of the drug from the balloon surface during the dilation procedure can be a good “gentle” alternative to the complicated procedure of transurethral injection. This can be achieved through the use of drug-eluting coatings. Note that the coating should not only hold the drug during the procedure of delivery, positioning, and inflation of the balloon in the BNC area but also ensure rapid drug release during dilation (several minutes). The choice of low-molecular-weight, highly water-soluble drugs for the coating is also preferred due to their high bioavailability and rapid diffusion into the tissue.

Microchamber arrays [12,13,14] are a modern, promising biomaterial for the encapsulation of low-molecular-weight, highly water-soluble substances that can be used as a drug-elution coating [15,16]. They are thin polymer films (from 1 to several microns) with many ordered, hermetically sealed microcontainers filled with cargo [17,18]. They can be easily manufactured from biocompatible polymers (polylactic acid (PLA) [19,20], poly (lactic-co-glycolic acid) (PLGA) [16,21], polycaprolactone (PCL) [16], and their copolymers). Microchamber arrays are formed by pressing together flat and embossed hydrophobic polymer films filled with the drug under slight heating (55–80 °C for 5–30 s). Using in vitro and in vivo models, it was proved that such a method of encapsulation allows retention of the biologically active properties of glutamic acid [22], nerve growth factor (NGF) [23], α-amylase [24], doxycycline [25], ceftriaxone [26], epinephrine (adrenaline) [21], and methylprednisolone [16]. It has also been shown that this biomaterial can release the drug both protractedly [16,19] and quickly (under external influences such as laser radiation [14,22,27,28,29] and high-intensity focused [19] and therapeutic [21,26] ultrasound). It opens up new approaches for personalized and custom-tunable therapy.

Here, we demonstrate the proof of concept that films with microchambers array based on PLA biopolymer can be used for the modification of a Foley catheter balloon surface for the addressed rapid release of Prednol-L (Figure 1B). Prednol-L is a commercially available and widely used drug consisting of methylprednisolone sodium succinate in the form of a lyophilizate and is widely used in clinical practice as a potent anti-inflammatory and immunosuppressive drug [30,31]. In this study, to the best of our knowledge, we explore for the first time the possibility of controlled drug release from microchamber arrays using diagnostic ultrasound equipment and compare the effectiveness of two different modalities of diagnostic ultrasound. The safety of the developed coating during balloon dilation of the bladder neck and urethra area is evaluated in vivo on rabbits.

## 2. Materials and Methods

### 2.1. Materials

Polylactic acid (PLA, 3 mm granule) was obtained from GoodFellow (Huntingdon, UK). Chloroform, 5(6)-carboxyfluorescein, glycerol, and sodium chloride were obtained from Sigma-Aldrich (Darmstadt, Germany). The deionized (DI) water (electric conductivity ~18.2 MΩ m^−1^ at 25 °C) for the solution preparation was obtained by the Milli-Q Plus185 from Millipore (Darmstadt, Germany). The anti-inflammatory drug, methylprednisolone sodium succinate (Prednol-L), was purchased from Gensenta (Istanbul, Turkey). The polydimethylsiloxane (PDMS) kit (Sylgard 184) was obtained from Dow-Corning (Midland, MI, USA).

### 2.2. Instruments

The Cobolt Tor XS (532 nm, 50 µJ, 1.9 ns) was used as a light source combined with a focusing lens (Olympus 4×/0.1 n.a.).

An Aixplorer ultrasound system (SuperSonic Imagine, Aix-en-Provence, France) with a convex probe XC6-1 and an SE12-3 MHz transrectal probe and an Epiq 5 (Philips Medical Systems B.V., Best, The Netherlands) C10-4ec MHz transrectal probe were used for triggered cargo release.

Scanning electron microscopy (SEM) measurements were performed with a VEGAIII (TESCAN, Brno, Czech Republic) microscope. The operating voltage was 5 kV. The gold was deposited onto the sample surface (~5 nm gold layer) using an Emitech K350 sputter-coater (Quorum Technologies Ltd., Ashford, UK).

The absorption spectra of Prednol-L were recorded using a TECAN Infinite M Nano + plate reader (TECAN, Männedorf, Switzerland) in standard 96-hole UV-transparent plates (Corning, NY, USA).

### 2.3. Fabrication of Patterned PDMS Stamp

The patterned PDMS stamp was preliminarily made based on a Kapton mold manufactured via the laser ablation method; 25 consecutive laser pulses were used to form one well. The resulting wells were in a cone form with a base diameter of 21 ± 1 µm, a height of 20 ± 2 µm, and an angle of ~35°. Spacing between the wells was set to 40 µm. The details of the PDMS casting and replication procedures are listed in [26].

### 2.4. Preparation of PLA Coating with Fluorescent or Biologically Active Cargo

Figure S1 shows the microchamber array fabrication process with SEM images of the resulting coating. The manufacturing routine of the film with microchamber arrays consists of several steps. At first, a PDMS stamp is dip-coated into the PLA–chloroform solution (1.5 wt%). The stamp was submerged into the solution and held in it for 10 s to ensure proper swelling. After that, the stamp was extracted with a constant velocity of 1 mm/s to form a uniform film on the PDMS surface.

In the second step, fine Prednol-L powder was loaded into the wells of embossed film. Excess powder removal was carried out using a brush.

In the third step, the resulting microcontainers were sealed by the heat-fusion of patterned and flat PLA films for 5 s at 80 °C. For this, a flat PLA film (3.5 wt% in chloroform) was preliminarily prepared on a glass slide using a thin-film applicator (Baker applicator gap 100 µm, 1 mm/s). After heat-fusion, the PDMS stamp was cooled to room temperature and detached from the film. The manufactured films with microchamber arrays measured 6 cm^2^ in area.

### 2.5. Coating Procedure of Foley Catheter Balloon

The balloon surface of a Foley catheter was cleaned with alcohol and dried using compressed air. A thin layer of Gluten BF-6 medical glue was applied to the balloon’s surface and left for 30 s. After the waiting period, a film with microchamber arrays with loaded Prednol-L was wrapped around the balloon and gently pressed against its surface.

### 2.6. Ultrasound-Induced Release of 5(6)-Carboxyfluorescein in Water and Gelatin Gel from a Foley Catheter Modified with PLA-Based Coatings with Microchamber Arrays Using a Diagnostic Ultrasound Device

A convex probe XC6-1 with an effective bandwidth of 1 to 6 MHz with gray-scale ultrasound imaging and shear wave elastography (SWE) imaging was used for the controlled rapid release of 5(6)-carboxyfluorescein from the Foley catheter modified with PLA-based coatings with microchamber arrays. The ultrasound settings were optimized for depth of penetration using an abdominal preset with a mechanical index (MI) of 1.5. Water and a hollow cylinder of 10% gelatin gel (ID = 10 mm, OD = 25 mm, length = 7 cm) were chosen as model media to demonstrate the release of fluorescein by ultrasound. Moreover, 2 mL of glycerol was added to 30 mL of gelatin gel to increase the elasticity of the phantom during balloon dilation. The fluorescent dye’s release from the coating was compared with and without ultrasound exposure (3 samples each) with an inflated balloon. The drug release in water was carried out at 37 °C.

### 2.7. Ultrasound-Induced Release of Prednol-L in Saline from a Foley Catheter Modified with PLA-Based Coatings with Microchamber Arrays Using Different Modalities of Diagnostic Ultrasound

Two different modalities of diagnostic ultrasound were used for the controlled rapid release of Prednol-L in saline from a Foley catheter modified with PLA-based coatings with microchamber arrays.

Conventional gray-scale ultrasound with shear wave elastography (SWE) was performed using a SE12-3 MHz transrectal probe with an effective bandwidth of 3 to 12 MHz. Ultrasound settings were adjusted for the penetration depth of a prostate preset with a mechanical index (MI) of 1.6.

Conventional gray-scale ultrasound was performed using a C10-4ec MHz transrectal probe with an effective bandwidth of 4 to 10 MHz. Ultrasound settings were adjusted for depth of penetration using a prostate preset with a mechanical index (MI) of 1.0.

The Prednol-L’s release from the coating was quantified with and without ultrasound exposure (6 samples each) with an inflated balloon. The drug release in saline was carried out at 37 °C.

### 2.8. Quantification of the Ultrasound-Induced Release of Prednol-L

To access the accelerated drug-elution under the influence of an US wave, Foley catheters were placed into polypropylene (PP) bags filled with 3 mL of saline. The inner volume of the PP bags was adjusted to ensure complete submersion of the expanded balloon under the saline solution. The released drug was quantified by absorption spectra.

### 2.9. In Vivo Study

All experiments were performed according to the relevant institutional (National Research Ogarev Mordovia State University, Saransk, Russia) and international regulations of the Geneva Convention of 1985 (International Guiding Principles for Biomedical Research Involving Animals). Animal ethics clearance was approved by the decision of the ethical committee (protocol No. 103 from 26 February 2022).

Adult male Soviet chinchilla rabbits were randomly assigned to 2 groups: implantation of Foley catheter without (control group, *n* = 3) and with (experimental group, *n* = 3) drug-eluting coating. The Foley catheters (6 Fr) were deployed transurethrally under general anesthesia and ultrasound guidance. Balloon dilation was performed at the border of the bladder neck and urethra using 1 mL of saline for 10 min. All animals were observed for any sign of deterioration in general health during the 24 h. Then, the rabbits were euthanized by anesthesia overdose.

The bladder neck and urethra were subjected to histological examination. Tissue samples were fixed in neutral formalin, desiccated using dehydrated isopropyl alcohol, and embedded in paraffin. The 5-μm-thick slides were stained with hematoxylin and eosin. Morphologic analysis of histological samples was performed using an Olympus digital image analysis system.

## 3. Results and Discussion

### 3.1. Ultrasound-Inducted Release of 5(6)-Carboxyfluorescein in Water and Gelatin Gel from a Foley Catheter Modified with PLA-Based Coatings with Microchamber Arrays Using a Diagnostic Ultrasound Device

At the first stage of the study, the 5(6)-carboxyfluorescein was used solely for the initial proof of concept for the controlled cargo release from microchambers under exposure to ultrasound in the diagnostic range as used in the clinic. In this regard, the ultrasound settings and exposure time (10 min [5]) were chosen according to the clinical procedure (balloon dilation). This allowed us to make no changes in the manipulation protocol.

For this, thin PLA films with ordered microchambers (cone with a diameter of 21 ± 1 µm, a height of 20 ± 1 µm, and an angle of ~35°) were produced (Figure 1C). A film with microchamber arrays was obtained by heat-fusing patterned and flat films together (Figure 1D). The patterned film’s thickness (0.42 ± 0.12 µm on its flat areas) was determined by the concentration of the initial PLA solution (1.5 wt%) and was optimized for this geometry of the PDMS matrix in our previous work [26]. In this work, it was shown that a film of this thickness better retained a low-molecular-weight and highly water-soluble drug such as ceftriaxone in comparison with a film obtained from a 1 wt% PLA solution. At the same time, this protocol provided a higher drug loading in comparison with the film obtained from a 2 wt% solution and remained sensitive to the therapeutic ultrasound exposure. The flat film had a significantly greater thickness—0.91 ± 0.11 µm.

**Figure 2:**
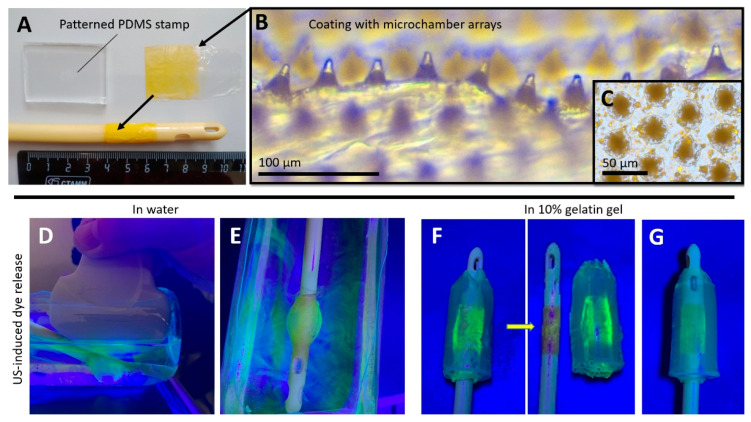
Typical photo of patterned PDMS stamp, coating with microchamber arrays loaded with 5(6)-carboxyfluorescein, and Foley catheter with the coated balloon (**A**). Optical images of the coating with microchamber arrays loaded with 5(6)-carboxyfluorescein (**B**,**C**). 5(6)-Carboxyfluorescein release during (**D**) and after (**E**) 10 min sonication of the inflated Foley catheter balloon with coating in water. 5(6)-Carboxyfluorescein release after (**F**) 10 min sonication of the inflated Foley catheter balloon with coating in 10% gelatin gel hollow cylinder. Gelatin gel hollow cylinder 10 min after balloon dilation without ultrasound exposure (**G**).

Crystals of 5(6)-carboxyfluorescein were loaded into the wells of the patterned film with a brush and heat-fused with a flat film. The color of the film changed to yellow due to the yellow powder loaded into it (Figure 2A–C). After this procedure, the film was fixed to the Foley catheter balloon using Gluten BF-6 medical glue (Figure 2A). Note that the PLA film on the upper part of the microchambers was thicker, which was well visualized after loading the microchambers using light microscopy (Figure 2B).

The coating showed good sensitivity to diagnostic ultrasound when a transabdominal convex probe XC6-1 with an effective bandwidth in the range of 1 to 6 MHz with gray-scale ultrasound imaging and shear wave electrography imaging was used. A part of the dye was already released in the water after balloon inflation, probably due to mechanical damage of the microchambers after the stretching. Controlled 5(6)-carboxyfluorescein release was observed during the entire ultrasound exposure time (10 min) on the coating in water (Figure 2D,E). An ultraviolet lamp was used to assist the clear visualization of the fluorescence dye’s release (Figure 2D–G).

Further, the experiment on the fluorescence dye’s ultrasound-induced release from the coating was carried out in a tissue phantom representing a hollow cylinder of 10% gelatin gel (Figure 2F and Figure S3). Here, local diffusion of the 5(6)-carboxyfluorescein into the gel over the entire coating area was detected. Note that, apart from robust triggering to open the microchambers, the ultrasound also facilitated the penetration of substances [32,33,34] and particles [35,36,37,38] into the biological tissues and structures. Balloon inflation without ultrasonic treatment did not lead to such visible gel staining (Figure 2G).

### 3.2. Ultrasound-Inducted Release of Prednol-L in Saline from a Foley Catheter Modified with PLA-Based Coatings with Microchamber Arrays Using Different Modalities of Diagnostic Ultrasound

At the next stage of the study, a quantitative analysis of the Prednol-L’s triggered release from the coatings using different modalities of diagnostic ultrasound was carried out.

**Figure 3:**
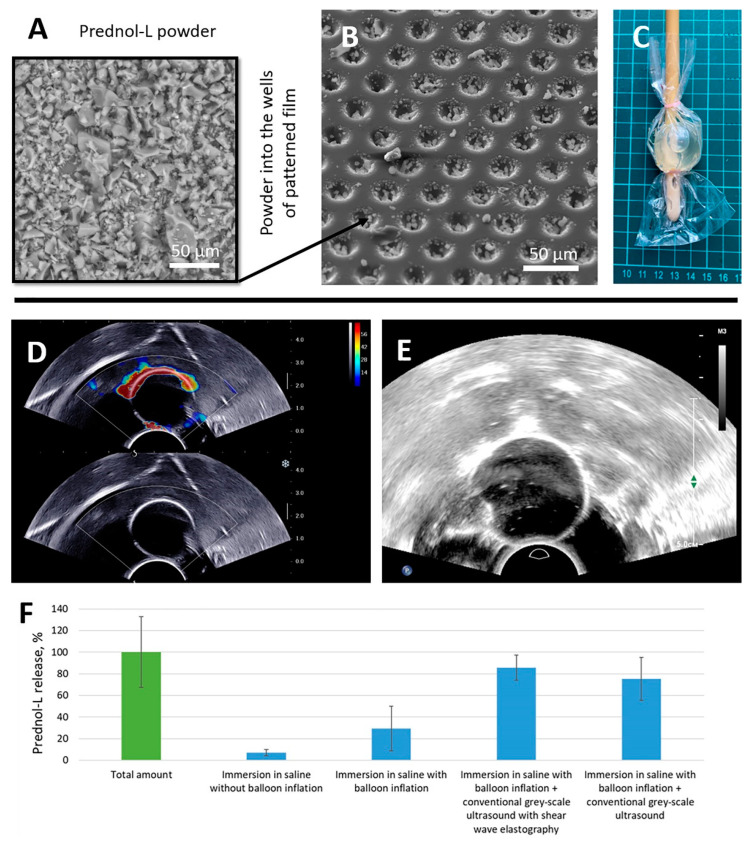
SEM image of Prednol-L powder after grinding in an agate mortar (**A**) and after filling into the wells of the patterned film (**B**). Foley catheter balloon placed in a sealed bag with 3 mL saline for release effectiveness assessment (**C**). Ultrasound imaging of Foley catheter balloon with coating obtained with transrectal probes with conventional gray-scale ultrasound regimen with (**D**) and without (**E**) SWE. Prednol-L release from the PLA-based coating with microchamber arrays imprisoned in a sealed bag with 3 mL saline with/without balloon inflation and with/without exposure of diagnostic ultrasound (**F**). Duration of ultrasound exposure—10 min.

Freeze-dried Prednol-L powder (Figure 3A) was ground in an agate mortar for 5 min and sifted through a stainless-steel mesh (50 µm). After this step, small crystals were loaded into the patterned film (Figure 3B). Patterned and flat PLA films were heat-fused together. The manufactured coating with the microchamber arrays was applied to 16 Fr Foley catheters; the coating contained 102 ± 33 µg of Prednol-L. The catheter’s balloon was packed in a polypropylene bag with 3 mL of saline (Figure 3C). The saline volume (3 mL) was selected to ensure complete submersion of the balloon and secure a detectable drug concentration. The packed catheter balloon was inflated with 10 mL of saline and placed in a large tank of water. The exposure of the conventional gray-scale ultrasound imaging with (Figure 3D) and without (Figure 3E) SWE regimen on the balloon surface led to the release of 87 ± 12 and 76 ± 20 µg of Prednol-L, respectively (Figure 3F). Thus, ultrasound exposure caused the release of a large fraction of packed drug, while no notable difference between ultrasound exposure regimes was observed. Immersion of the inflated and uninflated balloon in saline for 10 min without sonication also resulted in Prednol-L release but to a much lesser extent (only 30 ± 21 and 7 ± 3 µg, respectively).

A study of the Prednol-L release without ultrasound exposure from the developed coatings showed that approximately 90% of the load was released on the first day (Figure S2). In this regard, this type of coating is not suitable for the prolonged release of Prednol-L. At the same time, our previous results have shown that increasing the film thickness can prolong the release kinetics but with a decrease in the sensitivity of the microchamber arrays to ultrasonic exposure [26]. Thus, the variation of the film thickness and the shape of the chambers in a wide range allows the determination of the properties of the coatings, which will be optimal for specific clinical tasks.

Note that, in this study, transrectal probes were used and settings were adjusted for the penetration depth using a prostate preset. The duration of ultrasound exposure on the coating also coincided with balloon dilation duration, which brings our experiment as close as possible to the real clinical conditions of such manipulation. In general, this is promising for the use of the coatings for ultrasound-triggered release of Prednol-L in the BNC zone as an addition to the existing therapeutic strategy.

### 3.3. In Vivo Safety Assessment of Using a Foley Catheter Modified with an Ultrasound-Sensitive Coating Eluting Prednol-L

**Figure 4:**
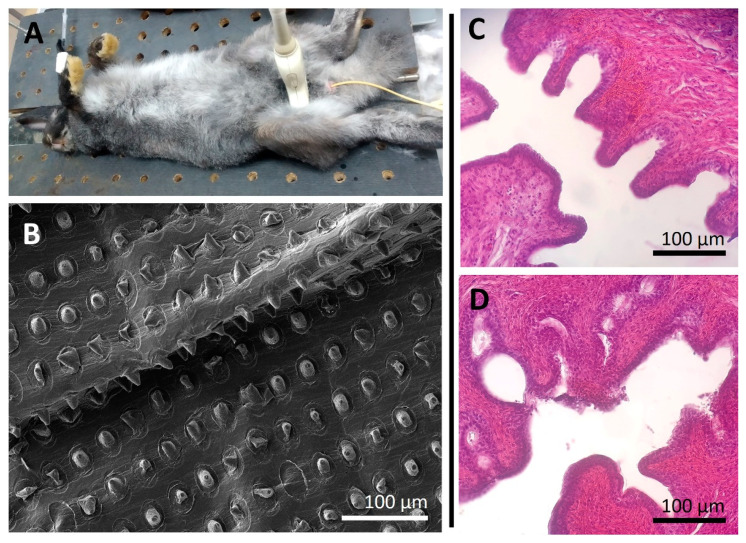
Typical photo of a rabbit during the balloon dilation using a Foley catheter (**A**). SEM image of PLA-based coating with microchamber arrays after balloon dilation (**B**). The morphological state of rabbit urethra 24 h after balloon dilation (during 10 min) using a Foley catheter (6 Fr) without (**C**) and with (**D**) a drug-eluting coating.

The safety of the developed coating’s application was studied on a rabbit model to explore the applicability of the method for in vivo research. These studies were conducted on healthy mature animals under general anesthesia. The uncoated and coated Foley catheters (6 Fr) were passed through the urethra in two groups of control and experimental animals under ultrasound guidance, respectively (Figure 4A). The catheter balloon was inflated in the bladder neck and urethra area using 1.5 mL of saline solution. Dilation lasted for 10 min. Ultrasound exposure to the balloon continued throughout the dilation period. Note that our therapeutic concept does not require additional manipulations from clinicians. The trigger for the opening of the microchambers is ultrasonic exposure, which is used during manipulation to position and guide the dilation process anyway [5].

After the procedure, the balloon was deflated, and the catheter was carefully removed from the urethra. Note that the coating remained securely attached to the catheter. which was extremely important to exclude the possibility of urethral blockage. Traces of blood on the coating were also not found during a routine examination of the catheter. This was probably due to the good elasticity of healthy tissues of the bladder neck and urethra but also indirectly indicated the absence of obvious tissue damage. Deterioration of the animal’s general condition and urination disorders during the next day after manipulation were also not observed.

SEM showed that the PLA-based coating did not change its morphological state significantly, although individual chambers were damaged (Figure 4B). Note, that the absence of obvious damage to the microchambers as seen in the SEM image does not mean that there is no release of the encapsulated substance. Our previous studies conducted using dextran-FITC as the model cargo showed cargo release from microchambers despite the absence of their obvious microdamage [21]. Possibly, the cargo release is carried out through smaller cracks in the PLA microchamber shell that are indistinguishable using SEM.

The folded surface of the mucous membrane of the urethra was observed in both cases (uncovered and covered catheter usage) during the histological analysis of tissues (Figure 4C,D). There were no foreign bodies, mucus clots, or epithelial cells in the lumen. Erythrocytes were visualized only in the capillaries of the submucosa and were absent in the lumen of the urethral canal. The mucosal epithelium was not interrupted. There were no hemorrhages in the submucosa and deeper layers. All this suggests that the insertion and removal of a polymer-coated catheter, dilation of the balloon, and ultrasonic exposure to the polymer coating do not lead to damage of the mucous membrane and stromal structures of the urethral canal.

In general, acute in vivo experiments demonstrated the absence of negative effects of using a coated Foley catheter for balloon dilation of the bladder neck and urethra area compared to an uncoated catheter.

## 4. Conclusions

A PLA film with microchamber arrays is a convenient and easily scalable biomaterial to accommodate and release pharmaceuticals. It is suitable for encapsulation and prolonged and/or controlled release of a wide range of substances. This paper presents a proof-of-concept usage of the microchamber arrays as a Foley catheter coating for triggered local delivery of an anti-inflammatory drug during balloon dilation of bladder neck contracture. The good sensitivity of the coatings to exposure to various types of ultrasound probes using different modalities of diagnostic ultrasound (gray-scale ultrasound imaging with and without SWE) was demonstrated. In the first stage, the possibility of triggered 5(6)-carboxyfluorescein release from microchambers into the water and gelatin gel under transabdominal convex probe exposure with gray-scale ultrasound imaging and SWE imaging was shown. In the second stage, the comparable efficiency of transrectal probes used in conventional gray-scale ultrasound with and without SWE regimen for triggered Prednol-L release was registered. Safety testing on rabbits showed the absence of negative effects of using a coated Foley catheter for balloon dilation of the bladder neck and urethra. Combining balloon dilation and triggered local delivery of anti-inflammatory drugs may become the “golden key” to significantly reducing the number of procedures required for bladder neck restenosis reduction and improving the quality of patients’ life.

## Data Availability

Data underlying the results presented in this paper are not publicly available at this time but may be obtained from the authors upon reasonable request.

## Supplementary Materials

The following supporting information can be downloaded at: https://www.mdpi.com/article/10.3390/pharmaceutics14102186/s1. Figure S1. The general scheme of preparation the microchamber arrays containing Prednol-L (A). SEM image of the free standing film with microchamber arrays based on PLA (B). Figure S2. Release profile of Prednol–L in saline (37 °C, 300 rpm) from PLA microchamber arrays. Figure S3. Ultrasound-induced release of 5(6)-carboxyfluorescein in gelatin gel from Foley catheter modified with PLA-Based coatings with microchamber arrays using diagnostic ultrasound devise.
