# Supplementary material for: Ultrasound-Triggerable Coatings for Foley Catheter Balloons for Local Release of Anti-Inflammatory Drugs during Bladder Neck Dilation
Source: Pharmaceutics. 2022 Oct 13;14(10):2186. doi: 10.3390/pharmaceutics14102186 (PMC9609387; doi:10.3390/pharmaceutics14102186)
Supplement: Supplementary file 1 [file pharmaceutics-14-02186-s001.zip › pharmaceutics-1929711-supplementary.pdf]

pharmaceutics

MDPI

Supplementary Materials

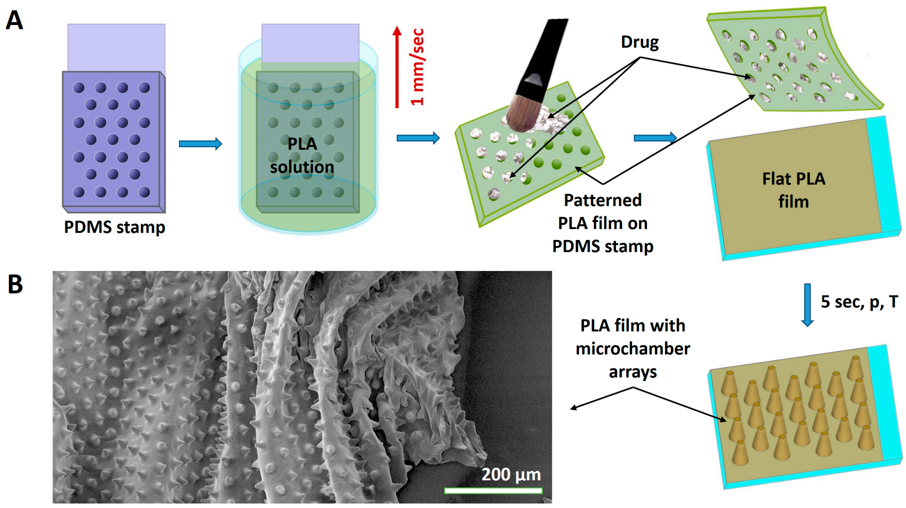

Figure S1. The general scheme of preparation the microchamber arrays containing Prednol-L (A). SEM image of the free standing film with microchamber arrays based on PLA (B).

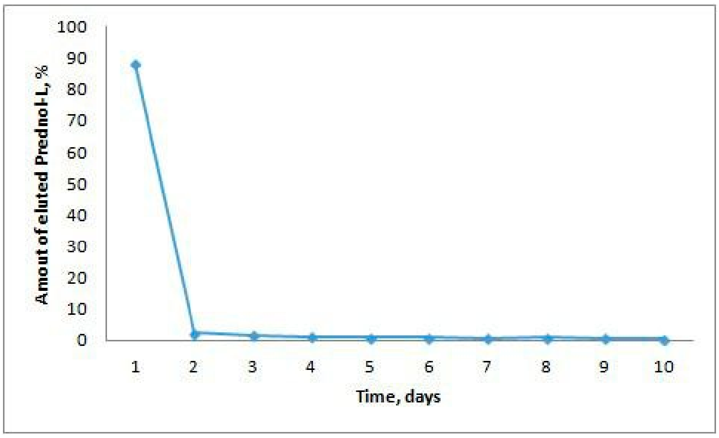

Figure S2. Release profile of Prednol – L in saline (37 °C, 300 rpm) from PLA microchamber arrays.

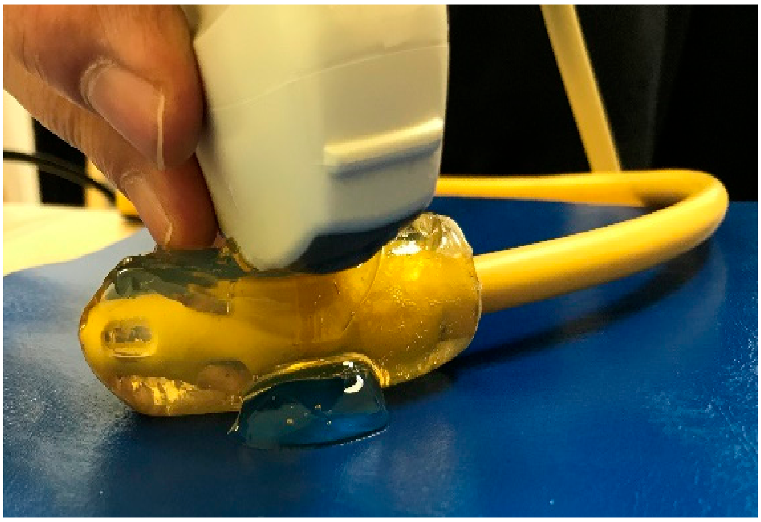

Figure S3. Ultrasound-induced release of 5(6)-carboxyfluorescein in gelatin gel from Foley catheter modified with PLA-Based coatings with microchamber arrays using diagnostic ultrasound devise.
